# Increased expression of MUSASHI1 in epithelial breast cancer cells is due to down regulation of miR-125b

**DOI:** 10.1186/s12860-021-00348-8

**Published:** 2021-02-04

**Authors:** Mahboobeh Forouzanfar, Liana Lachinani, Kianoush Dormiani, Mohammad Hossein Nasr-Esfahani, Kamran Ghaedi

**Affiliations:** 1 Department of Cell and Molecular Biology and Microbiology, Faculty of Biological Science and Technology, University of Isfahan, Hezar Jerib Ave., Azadi Square, Isfahan, P.O. Code 81746 Iran; 2 Department of Animal Biotechnology, Cell Science Research Center, Royan Institute for Biotechnology, ACECR, Isfahan, P.O. Code 816513-1378 Iran

**Keywords:** Breast cancer, Epithelial markers, microRNA, Musashi1

## Abstract

**Background:**

Musashi1 (MSI1) is an oncogenic protein with a crucial role in the proliferation and characteristics of the epithelial cells in breast cancer. The change in expression of *MSI1* has a role in solid tumor progression. There are different factors that regulate *MSI1* expression in various cancer tissues including microRNAs which are considered as one of the most important of these factors. The aim of our study is identification of the molecular cause of maximal expression of *MSI1* in epithelial breast cancer cell lines.

**Results:**

Among predicted microRNAs, miR-125b, miR-637 and miR-802 were able to significantly reduce the luciferase activity. In addition, the relative expression of these three miRNAs were measured in the cancerous cell lines that results showed a significant reduction in expression of all microRNAs. On the other hand, only the overexpression of miR-125b caused a change in the expression pattern of *MSI1* in breast epithelial cancer cell lines*.* Accordingly, our results demonstrated that the exogenous expression of miR-125b decreased not only the MSI1 protein but also expression of epithelial markers in breast cancer cells.

**Conclusions:**

The results of luciferase reporter assay showed that *MSI1* is a direct target for miR-125b in epithelial breast cancer cells. Moreover, higher amount of MSI1 in those cell lines seems due to the reduced amount of miR-125b, which is responsible for epithelial features of those kinds of cancer cells. Therefore, the modulation of miR-125b may be a potential approach to help to combat against epithelial breast tumors.

**Supplementary Information:**

The online version contains supplementary material available at 10.1186/s12860-021-00348-8.

## Background

Breast cancer has the highest incidence of female cancers worldwide. Analysis of global gene expression and DNA microarrays from cancer tissues asserted that a variety of genes are causative for this type of cancer. Accordingly, breast cancer is divided into five groups; a) HER2-positive, b) luminal A, c) luminal B, d) basal-like and e) normal-like subtypes [1].

In mammalian cells, the family of musashi (MSI) as RNA-binding proteins are highly conserved and have two members, musashi1 (MSI1) and musashi2 (MSI2). MSI1 has a main function in self renewal and maintenance of stem cells [2, 3]. MSI1 increases proliferation of mammary stem cells through triggering of two signaling pathways; Notch and Wnt [4]. Moreover, *MSI1* is strongly expressed in multiple solid tumors such as brain [5–7], lung [8], ovary, squamous oral, colorectal and renal cancers [9–12]. On the other hand, MSI2 is reported to be upregulated in hematopoietic cancer cells [13]. The expression of MSI1 in HER2-positive breast cancer cell lines is correlated with HER2 and knock down of *MSI1* reduces colony expansion of spheroid cultures in tumor cells [14]. Furthermore, MSI protein is essential for the preservation of epithelial property of breast cancer cells whereas overexpression of *MSI2* regulates in vivo processing of epithelial-to-mesenchymal transition (EMT). Conversely, knocking down of *MSI1* or *MSI2* in the BT474 cell line enhances mesenchymal markers and decreases epithelial markers expression [15].

MicroRNAs belong to noncoding RNAs that control gene expression mostly through binding to 3’UTR of target genes. Generally, miRNAs regulate target gene expression via reduction of mRNA stability or translation [16–18]. MicroRNAs have diverse roles in various steps of breast cancer progression, which were indicated in recent studies [19–21]. 3`-untranslated region (3’UTR) of *MSI1* has a length of 1800 nucleotides and is predicted to contain different sites for binding of endogenous miRNAs. The documentation shows that *MSI1* has an oncogenic function in breast cancer progression, therefore any modulation in *MSI1* expression could be considered as a possible therapeutic approach against breast cancer progression. Thus, the aim of our study was identification of putative miRNAs, which modulate *MSI1* transcript in breast cancer cells. For this purpose, we first predicted the miRNAs that bind to 3’UTR of *MSI1* using bioinformatic databases and then confirmed the direct binding of selected miRNAs to *MSI1* 3’UTR by luciferase assay. Finally, overexpression of miR-125b in human breast epithelial cancer cells modulated the expression of *MSI1* as well as epithelial markers.

## Results

### Up-regulation of *MSI1* was only detected in epithelial breast cancer cell

**Fig. 1:**
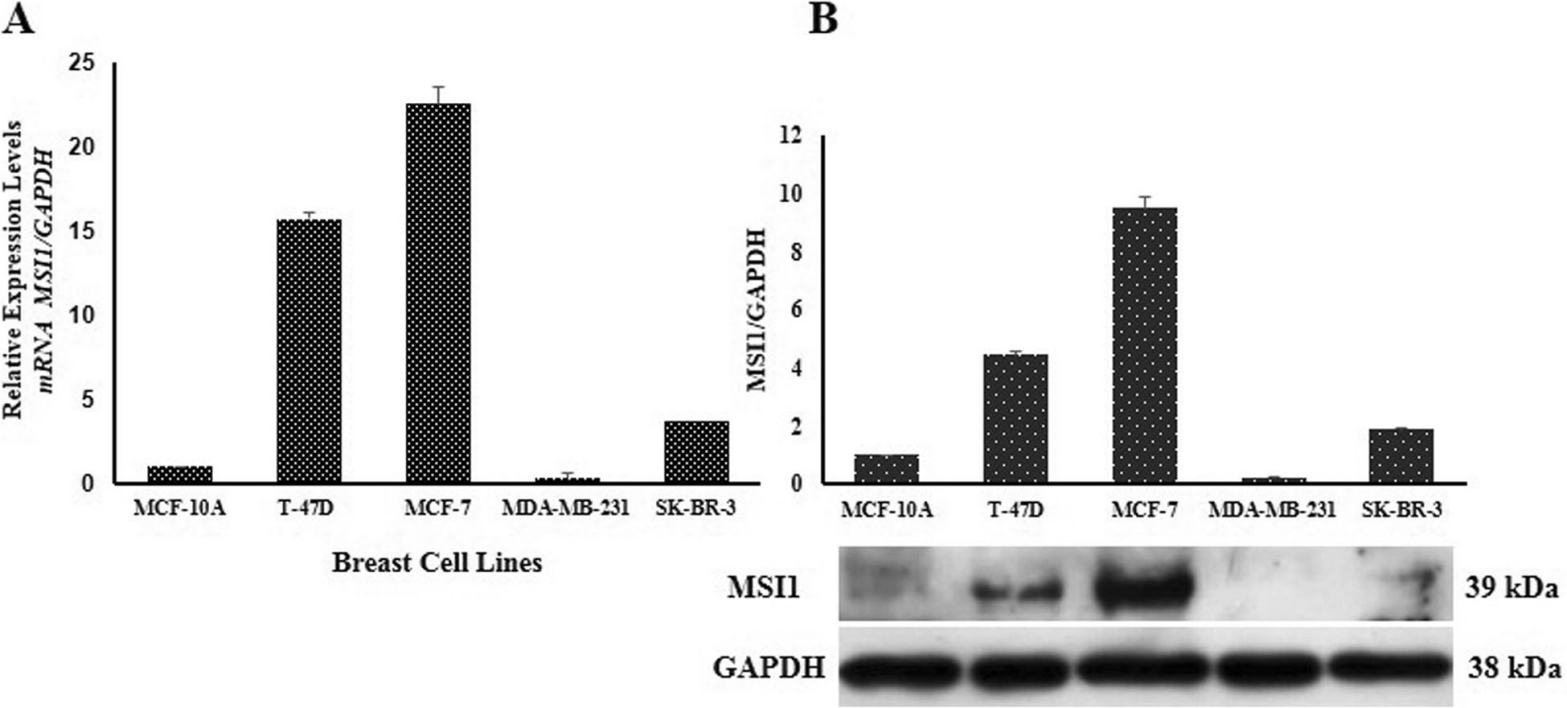
*MSI1* expression and western blot analyzing in breast cancer cell lines. **a** *MSI1* expression was measured with real-time PCR. **b** MSI1 protein expression was analyzed using western blotting. Each expression was quantified and normalized with *Gapdh*. Represented value bars are the mean of triplicate independent experiments ± SEM

Analysis of transcript and protein of MSI1 were carried out in several breast cancer cell lines with different origins. The results demonstrated that MSI1 was highly expressed in T-47D and MCF-7 cells (epithelial cell line) but not in SKBR3 (HER2-positive cell line) and MBA-MD-231 cells (mesenchymal cell line) (Fig. 1).

### Bioinformatics prediction of microRNAs targeting *MSI1*

**Fig. 2:**
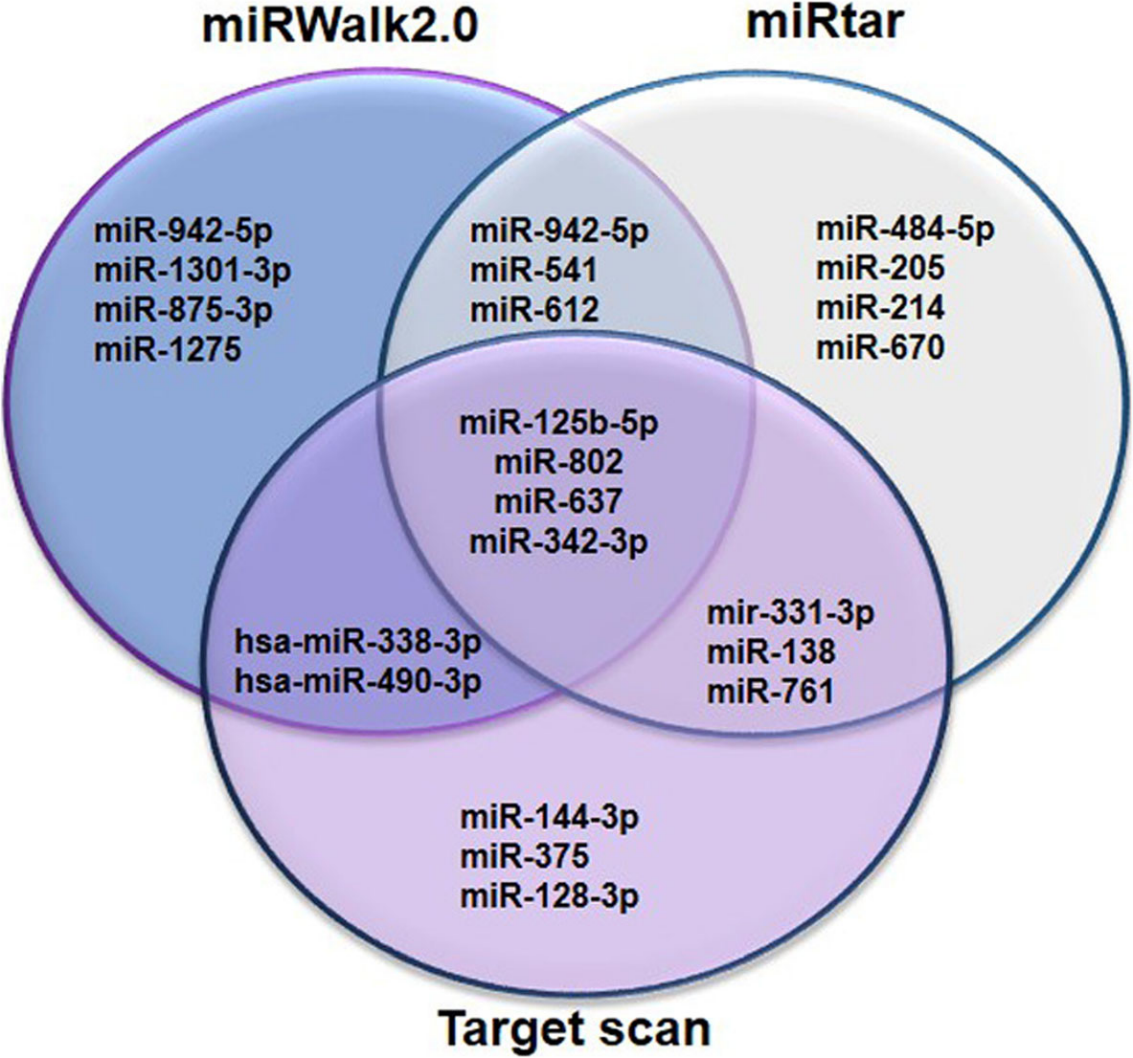
Schematic representation of bioinformatic sites for miRNAs prediction. For prediction of microRNA target sites, different scores in bioinformatic sites were used

For prediction of those microRNAs which target *MSI1*, the following conditions were considered: the minimum folding free energy, context++ score, conservation, number and site seed type of miRNA binding to *MSI1* 3′-UTR [22]. Due to maximal expression of *MSI1* in MCF-7 and T-47D cancer cell lines (epithelial cell lines) compared to MCF-10A (normal breast cell line), the levels of predicted miRNAs were assessed using a GEO dataset GSE70480, which was related to the expression profile of miRNAs in aforementioned cell lines. Based on extracted data, miR-125b-5p, miR-802, miR-490-3P, miR-338-3p and miR-637 were selected due to their effect on *MSI1* downregulation (Fig. 2, supplementary Figure 1). On the other hand, miR-342-3p was excluded from the list as it had previously reported that its expression was high in MCF-7 similar to what reported for *MSI1.* Therefore, miR-342-3p could not be supposed to target *MSI1* [23]. Also, the selection of miR-802 for further analysis was due to reduced expression in MCF-7 compared to normal breast cell line [24].

### Down-regulation of predicted microRNAs were verified in epithelial breast cancer cells

**Fig. 3:**
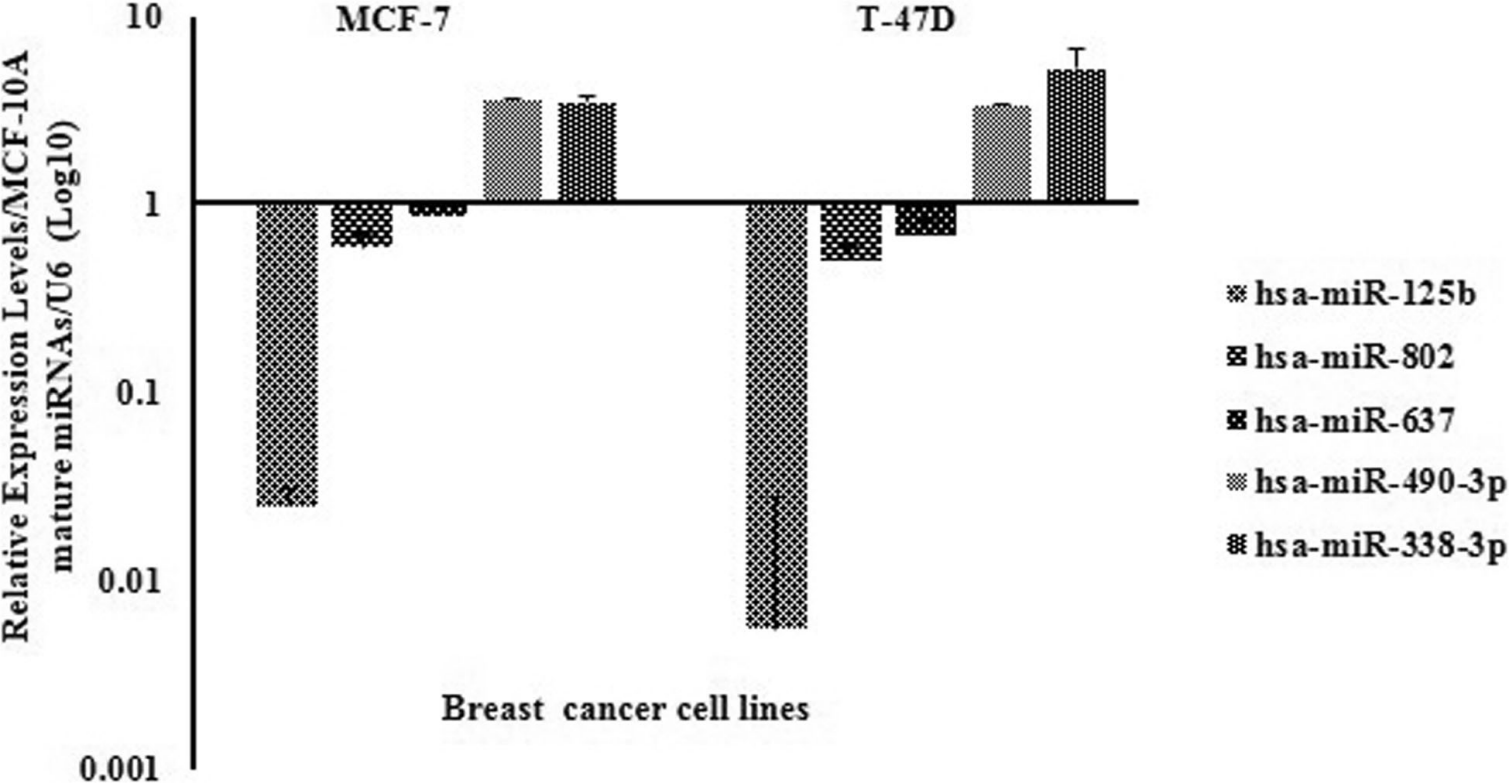
Expression of mature miRNAs in breast cancer cell lines in comparison of normal breast cell line, MCF-10A. Mature miRNAs expression were measured with real-time PCR. Expression of U6 was used for quantification and normalization of the results. Represented value bars are the mean of triplicate independent experiments ± SEM

Since expression of *MSI*1 was high in both epithelial cancer cells (T47-D and MCF-7), the levels of predicted mature miRNAs were assessed in those cell lines (Fig. 3). Mature miRNAs expression were measured with MiRCURY LNA primer mix (Exicon, USA) and normalized with MiRCURY LNA U6 (Exicon, USA). However among them, only miR-125b-5p, miR-637 and miR-802 were selected for luciferase reporter assay because these microRNAs had lower expression values than others in both epithelial cancer cells compared to MCF-10A.

### Confirmation of direct interaction of selected microRNAs with *MSI1* transcript

**Fig. 4:**
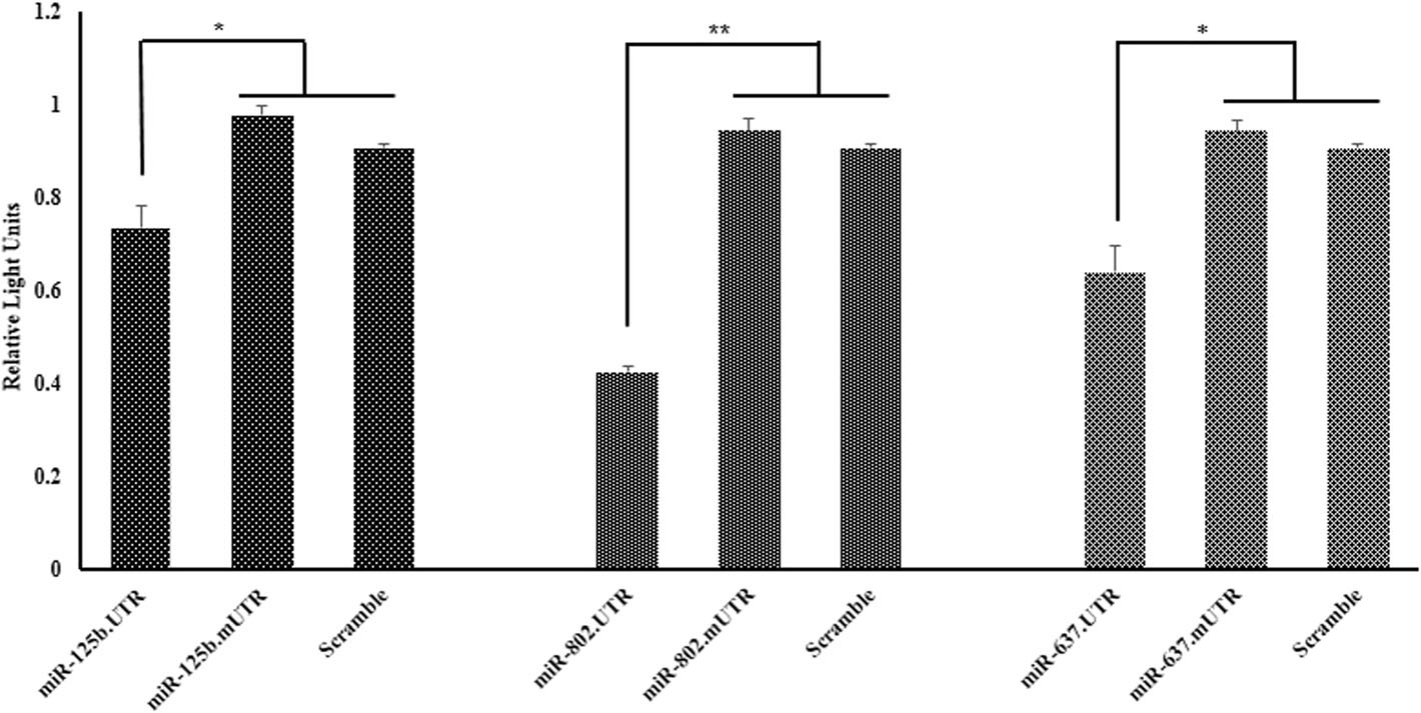
Luciferase activity assay after overexpression of miRNA precursors. HEK393T cells were co-transfected with recombinant plasmids, which included *MSI1* 3’UTR downstream humanized *Renilla* luciferase reporter gene (hRluc) and miRNAs precursors. Also, HEK393T cells co-transfected with mutated Msi1 3’UTR for each miRNAs binding site downstream of hRluc luciferase reporter gene. For negative control analysis, HEK393Tcells co-transfected with a scrambled negative control miRNA (Control miRNA) and wild Msi1 3’UTR downstream hRluc. Then luminescence was measured. Error bars denote S.E.M. for three independent repeats in each experiment. Independent student *t*-test indicated differences between (*p < 0.05; **p < 0.01)

To confirm direct binding of miR-125b-5p, miR-637 and miR-802 to 3′-UTR of MSI1 transcript (Supplementary Table 1), HEK293T cells were transfected with plasmids expressing precursors of both microRNAs as well as wild type or mutated MSI1 3’UTR. Transfected cells were harvested, lysed and the cell lysates were used for dual-luciferase reporter assay. Consistent with bioinformatics data, selected microRNAs significantly repressed luciferase activity. Elimination of microRNAs binding sites from 3’UTR of *MSI1* resulted in significant increase in luminescent signals (Fig. 4). Therefore, direct targeting of *MSI1* transcript by those microRNAs was verified.

### Elevated amount of miR-125b was able to inhibit *MSI1* expression in cancer cells

**Fig. 5:**
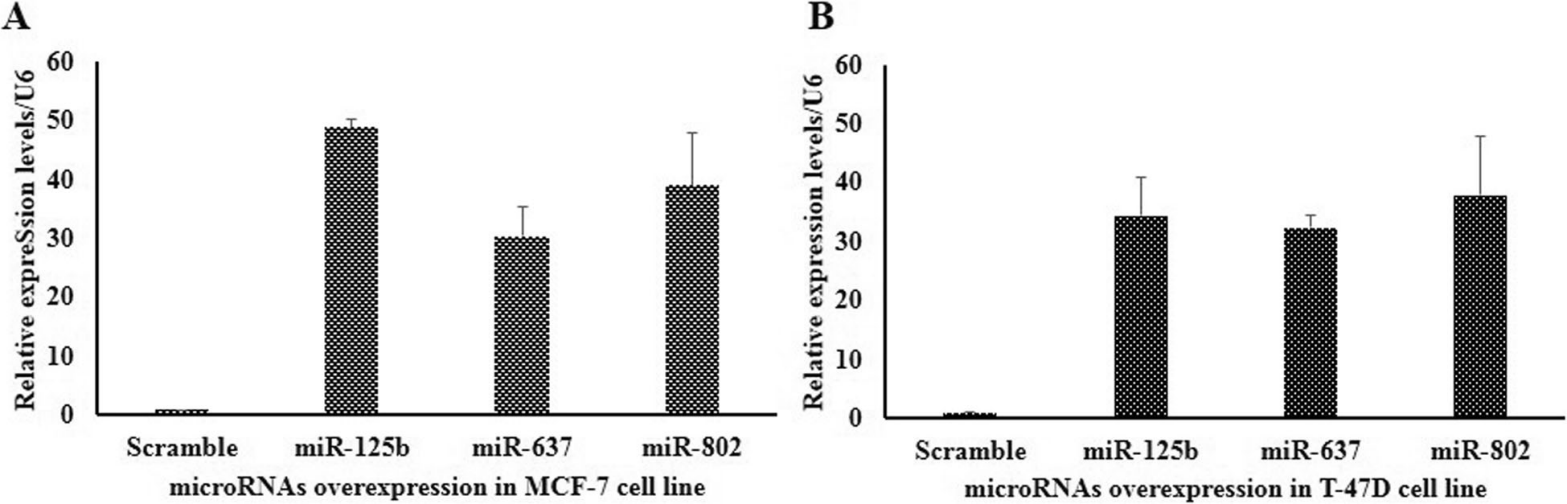
Expression of mature miRNAs in breast cancer cell lines after transfection of recombinant plasmids [pBUD/precursor miRNAs expression plasmids] in MCF-7 and T-47D cell lines

**Fig. 6:**
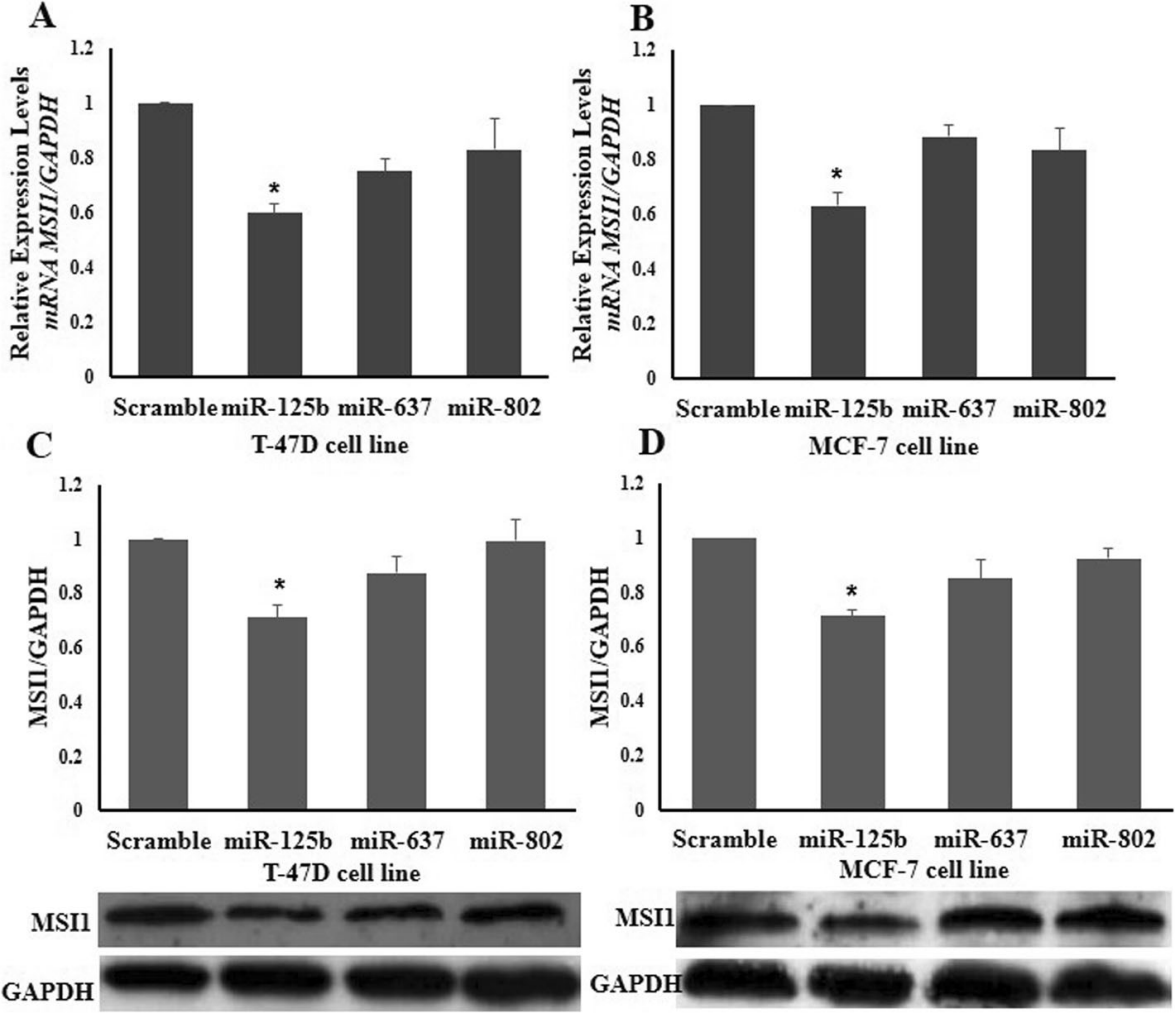
*MSI1* mRNA and protein expression analyzed in breast cancer cell lines. *MSI1* expression analyzed in (**a**) MCF-7 and (**b**) T-47D breast cancer cells transfected with miRNA precursor plasmids and compared to cells transfected with a scrambled negative control miRNA. **c** MSI1 protein expression analyzed in MCF-7 and (**d**) T-47D breast cancer cells transfected with miRNA precursor plasmids as compared to cells transfected with a scrambled negative control miRNA (Control miRNA). Error bars denote S.E.M. for three independent repeats in each experiment. The expression of mRNA was analyzed using RT-qPCR, and normalized to *GAPDH* in comparison to untransfected cells. MSI1 protein expression was analyzed using western blotting Error bars signify S.E.M. for three independent repeats in each experiment. One-way ANOVA indicated differences (*p < 0.05)

To evaluate which one of microRNAs has a potential to negatively modulate the expression of *MSI1* in epithelial cancer cells, MCF-7 and T-47D cells were transfected with plasmids expressing miRNA precursors. Expression of mature miRNAs in breast cancer cell lines after transfection were analyzed (Fig. 5). According to our data, ectopic expression of miR-125b reduced MSI1 transcript (Fig. 6 A, B) and its protein compared to negative control (Universal scramble) in both cell lines (Fig. 6 C, D). Surprisingly, in contrast to luciferase data, overexpression of miR-637 and miR-802 were not able to decrease the *MSI1* expression in breast cancer cells. Consequently, we decided to use miR-125b for evaluating the effect of the microRNA on expression of epithelial markers in breast cancer cells.

### Ectopic expression of miR-125b reduced epithelial markers

**Fig. 7:**
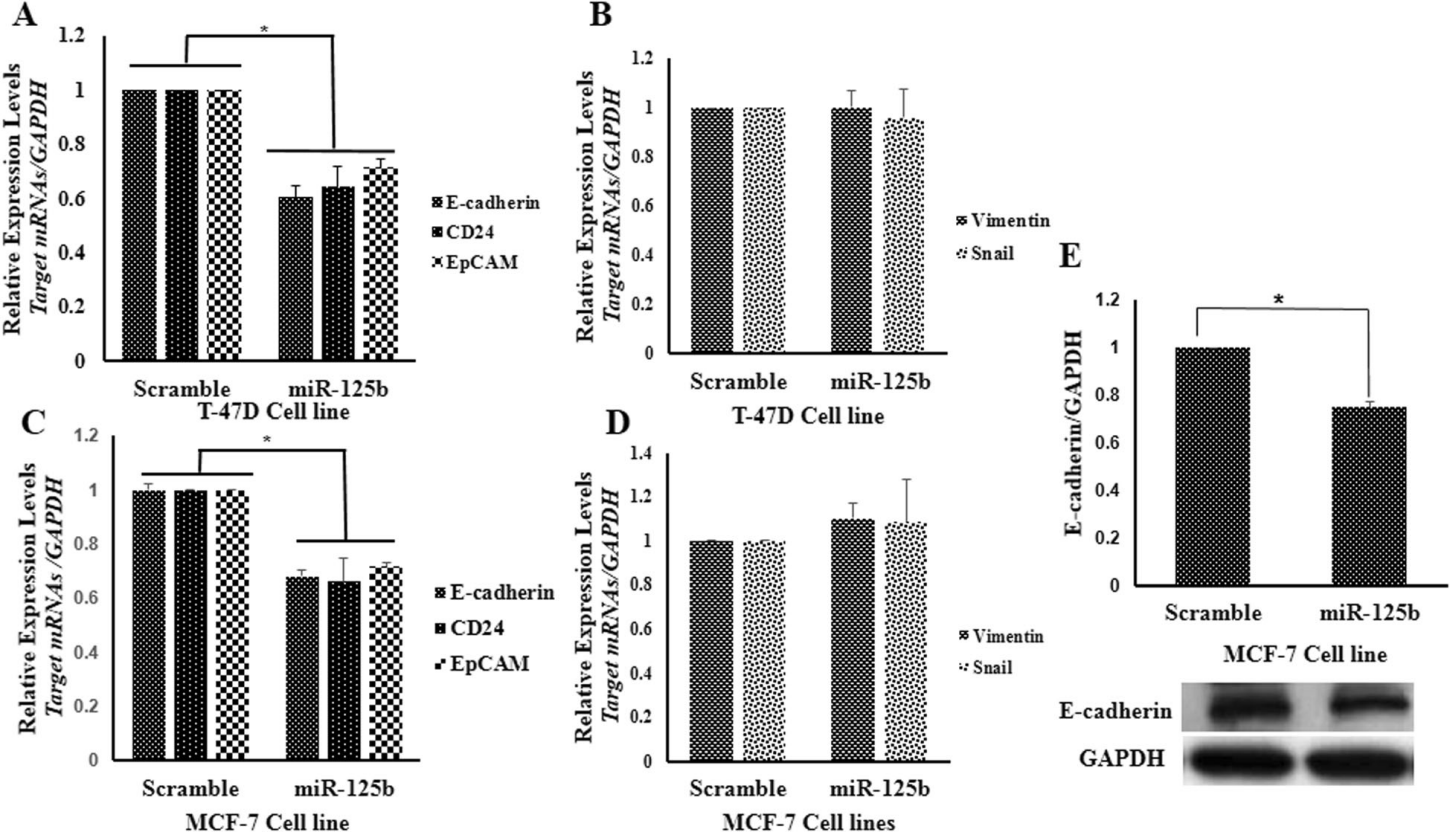
Analysis of epithelial and mesenchymal expression markers. **a** Epithelial marker expression in T-47D cells. **b** Expression of mesenchymal marker in T-47D cells. **c** Expression of Epithelial marker in MCF-7 cells. **d** Expression of mesenchymal marker in MCF-7 cells. **e** Western blot analysis for E-cadherin in MCF-7 cell lines. Error bars denote S.E.M. for three independent repeats in each experiment. Data were analyzed with the independent-samples *t*-test (*p < 0.05)

**Fig. 8:**
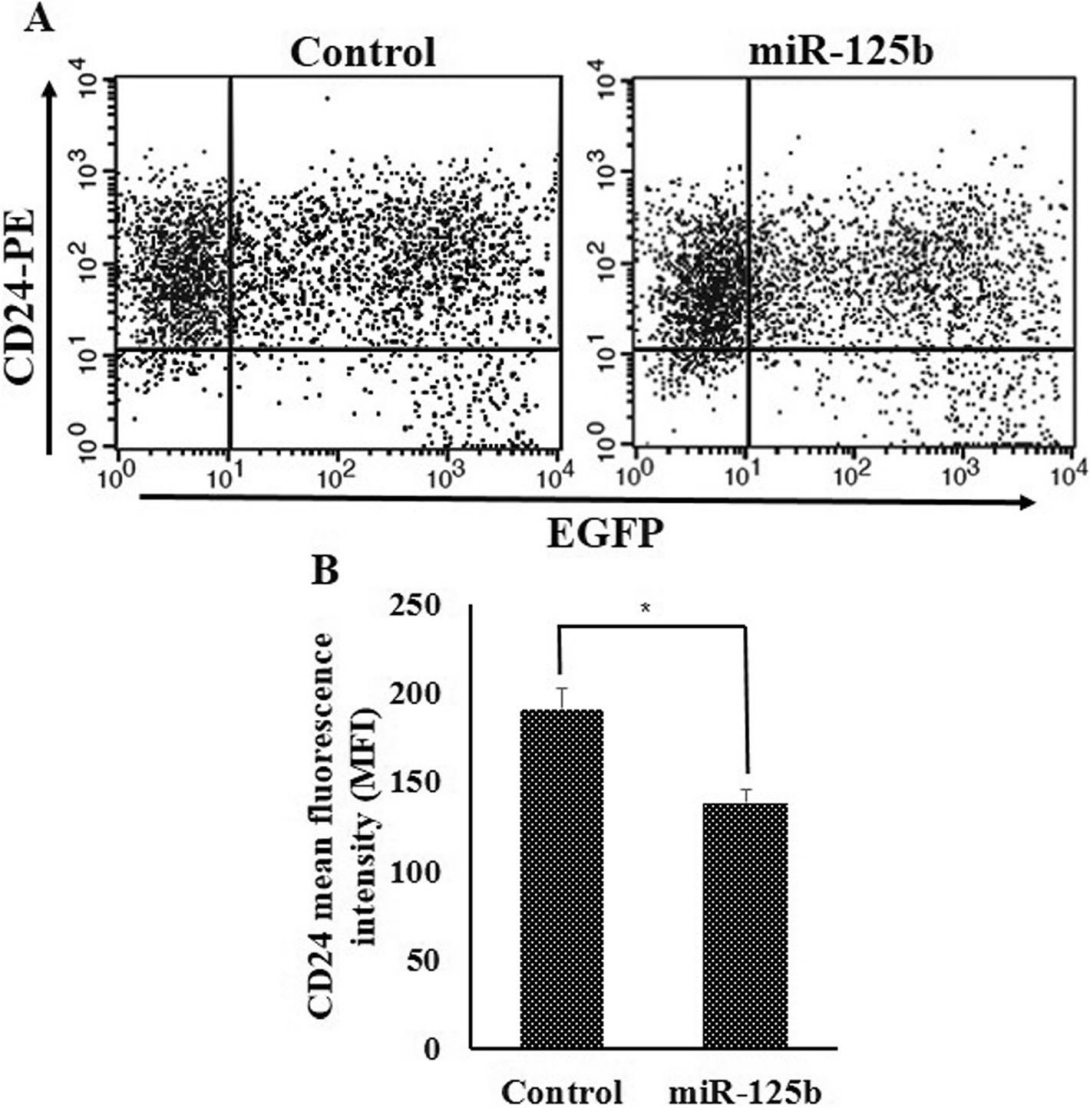
Analysis of CD24 expression through Flow cytometry. **a** Flow cytometry analysis of CD24 in EGFP positive cells showed that overexpression of mir-125b reduced the intensity of CD24 in MCF-7 cells. **b** Graphs of quantification of CD24 intensity by flow cytometry. Error bars denote S.E.M. for three independent repeats in each experiment. Data were analyzed with the independent-samples *t*-test (*p < 0.05)

**Fig. 9:**
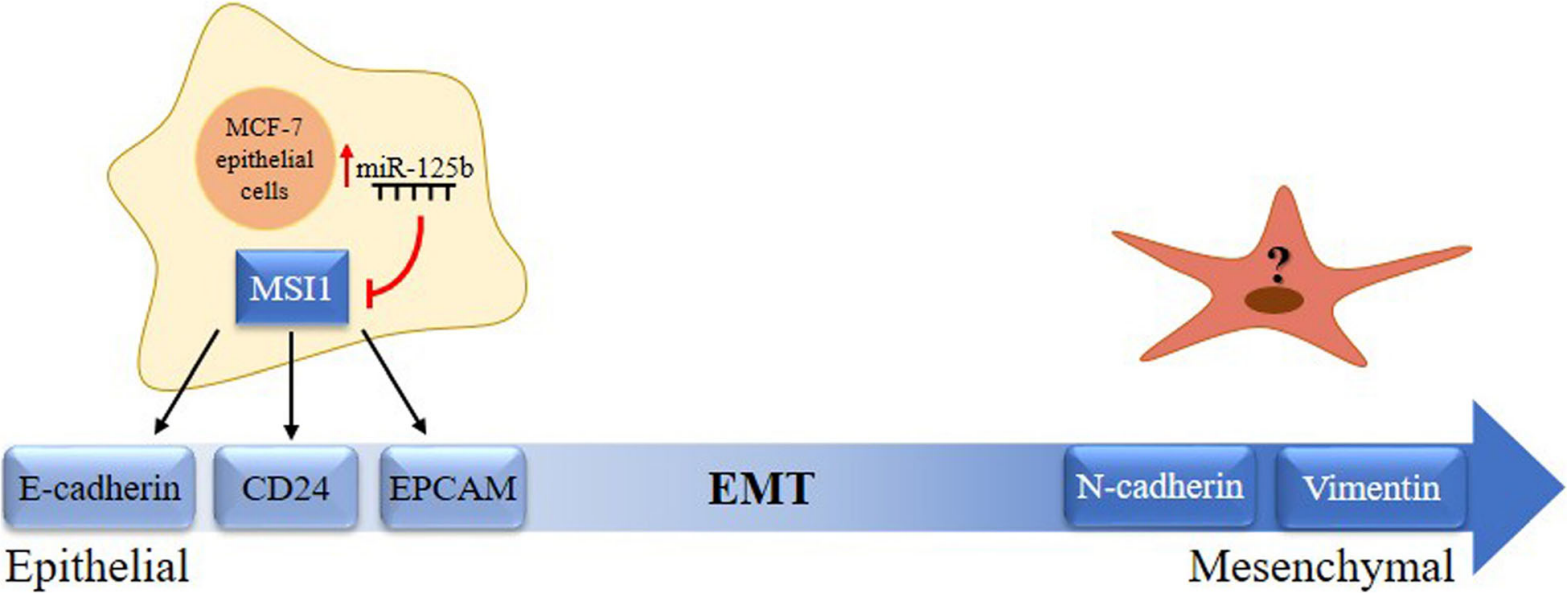
Schematic representation of MSI1 regulation through overexpression of miR-125b in epithelial–luminal state of breast cancer cell lines. Overexpression of miR-125b in human breast epithelial cancer cells modulated the expression of *MSI1* as well as epithelial markers

In previous studies, it was shown that knockdown of *MSI1* in epithelial cells such as BT474 was responsible for reduction in epithelial markers including E-cadherin and epithelial cellular adhesion molecule (EpCAM) and CD24 [15]. Inversely, there was an increase in mesenchymal markerssuch as Fibronectin and jagged1 in similar circumstance. Our data were evident that ectopic expression of miR-125b in MCF-7 and T-47D cell lines gave rise to a reduction in E-cadherin, EpCAM and CD24 (Fig. 7a, c). However, increasing expression rate of miR-125 could not able to change the expression of mesenchymal markers (Fig. 7b, d). Moreover, Immunoblotting of E-cadherin in MCF-7 cells confirmed the reduction of epithelial markers followed by ectopic expression of miR-125b (Fig. 7e). Change of CD24 expression in EGFP positive population cells, which were transfected with miR-125b.pBUD and Usc. Pbud were evaluated by flow cytometry. A transfection efficiency of 30% was obtained in MCF7 cells (supplementary figure 2). Analysis of fluorescence intensity revealed that expression of CD24 was reduced after overexpression of miR-125b (Fig. 8). Reduction of MSI1 intensity in EGFP positive cell population in MCF-7 cells transfected with miR-125b.pBUD were shown in supplementary figure 3. Also, MCF-7 cells were transfected with EGFP.pBUD plasmid (USc.pBUD) as negative control. EGFP positive and negative cells have the same intensity for MSI1 (supplementary figure 4). Flow cytometry results were shown in supplementary figure 5. Eventually, according to the results, we concluded that down-regulation of the miR-125b is responsible for uprising of MSI1, which is crucial for maintenance of the epithelial phenotype in breast cancer cells (Fig. 9).

## Discussion

RNA binding protein, MSI1, is highly expressed in different solid tumors [25, 26]. MSI1 has a crucial role in stem cell and cancer stem cell proliferation [27, 28]. MSI1 protein and notch signaling pathway are two key regulators for asymmetric division of the stem cell population in mammary epithelial cells [29]. In breast cancer, a positive correlation between expression of *ErbB2* and *MSI1* was observed. It has demonstrated that *MSI1* expression is increased in spheroid culture derived from breast cancer lines such as T-47D and MCF-7 [14]. In breast cancer, MSI1 has also a critical role in EMT. Hence expression of MSI1 in T-47D and MCF-7 [epithelial cell lines] was significantly higher than MDA-MB-231 [mesenchymal cell line].

MicroRNAs regulate gene expression at post transcriptional levels. Recent data have shown that miR-802 as a tumor suppressor could control proliferation of MCF-7 cells through expression regulation of *FOXM1* [24]. Similarly, miR-637 has also antitumor activity, such that its expression was reduced in hepatocellular carcinoma cell line (HCC) and clinical specimen of cancer patients (reference). The experiments revealed that overexpression of miR-637 in some hepatocellular carcinoma cell lines gave rise to a reduction in proliferation of cancer cells by regulation of signal transducer and activator of transcription 3 (STAT3) [30]. Furthermore, it was indicated that miR-637 has a tumor suppressor role, which prevents migration and invasion of cancer cells by suppression of AKT1 in human glioma [31]. Recently, it was reported that overexpression of miR-125b decreased the proliferation of breast cancer cells and promoted their apoptosis by regulation of G2/M phase. miR-125b controls cell cycle through regulation of a number of target genes such as *multiple EGF-like-domains 9* (*MEGF9*), *cyclin J* (*CCNJ*), *aminopeptidase A* (*ENPEP*) and *casein kinase 2-alpha* (*CK2-α*) [32]. Likewise, it was reported that ectopic expression of miR-125b could prevent migration and proliferation of breast cancer cells through regulation of KIAA1522 [33].

The long 3’UTR fragment of *MSI1* coding sequence has several potential binding sites for microRNA. In the present research, utilizing in silico analysis of a GEO dataset (GSE70480), we predicted several miRNAs that were able to bind 3’UTR of *MSI1*. Expression of the predicted miRNAs (miR-125b-5p, − 802, − 637, − 490-3p and − 338-3p) was assessed in some epithelial cancer cell lines in which *MSI1* expression was high. Amongthem, three miRNAs (miR-125b-5p, − 802, and − 637) with low expression rate in tested breast cancer cells were chosen for further investigation. All of three selected miRNAs were shown to bind directly to *MSI1* 3’UTR as verified by luciferase reporter assay. Among the aforementioned miRs, miR-125b was able to regulate *MSI1* expression whereas overexpression of miR-637 and -802 could not able to modulate transcript of *MSI1* in MCF-7 and T-47D cells. Our further in silico analysis using RNA-binding protein-specific database (http://rbpdb.ccbr.utoronto.ca/) indicated that RNA binding motif protein X-Linked (RBMX) could attach to the same sequence of *MSI1* 3’UTR, which covers the miR-637 binding site. Interestingly, RBMX are actively expressed in breast cancer cells according to the human protein atlas data (https://www.proteinatlas.org) [34, 35]. In this respect, several independent studies have indicated that there is a counteraction between RNA binding protein and miRNAs for accessibility to 3’UTR of target genes [36, 37]. Therefore, it is more likely that RBMX compete with miR-637 for binding to its target sequence in MSI1 3’UTR. However to validate such hypothesis, further analyses are needed to be performed. On the other hand, following down regulation of *MSI1* by miR-125b MCF-7 and T-47D cell lines, the expression of epithelial markers including *E-cadherin*, *CD24* and *EpCAM* was also diminished. But conversely, transcript level of mesenchymal marker (*Vimentin*) was not affected in these cell lines, presumably due to the lack of enough expression of *Vimentin* as reported previously [38].

However, our data were in good agreement with the recent study that down regulation of *MSI1* in epithelial cell line, BT474, caused a reduction in epithelial markers [15]. It seems that this effect is cell line dependent, as transfection of miR-125b inhibitor in triple negative breast cancer lines (TNBC) was responsible for reduction in cellular proliferation and process of EMT [39]. In these lines such as MDA-MB-468 and MDA-MB-231, the cells are negative for estrogen receptors, progesterone receptors, and excess HER2 protein, [39]. Similarly, in an agreement to our data, overexpression of miR-125 in Hs578T cells inhibited the EMT through a reduction in *MAP 2 K7* [40].

## Conclusions

Collectively, we indicated that *MSI1* is a potential target of miR-125b. Hence, miR-125b could be considered as a potential factor for regulation of the progress of a number of breast cancer types and is potentially applicable for therapeutic purposes. However the exact mechanism underlying this phenomenon should be investigated in further studies as it would need to be critically looked at while comparing the same phenomenon with primary cells.

## Methods

### Bioinformatics sites for miRNAs prediction

In order to predict the miRNAs interact with *MSI1* RNA, several bioinformatics database were implemented as Target Scan (targetscan.org), PicTar (pictar.mdc-berlin.de/cgi-bin/PicTar_vertebrate.cgi), miRWALK (mirwalk. Uni-hd. de/) and miRTar.human (http://mirtar.mbc.nctu.edu.tw/human).

### Construction of expression plasmids

For construction of the plasmids expressing human miRs-802, − 637, −125b, miRNA precursors were PCR amplified by specific primers (Table 1, A) and using human genome as a template. Then, the desired sequences were cloned into *Sal*I and *Xba*I sites of pBUD4.1 expression plasmid under CMV promoter. All restriction enzymes were obtained from Thermo Scientific, USA. To verify the expression of the constructs, the recombinant vectors were double digested with *Sal*I and *Xba*I restriction enzymes. The recombinant plasmids were confirmed by restriction digestion (Supplementary figure 6). For negative control plasmid, universal scramble sequence was cloned into EGFP.pBUD plasmid (USc.pBUD) as reported previously [41]. Schematic representation of recombinant plasmids are displayed in supplementary figure 7. For Luciferase reporter assay, the multiple cloning site in pSICHECK2 plasmid (Promega, USA) was modified by cloning a synthetic DNA fragment into *Sal*I and *Not*I sites in multiple cloning site and resulted vector was named as pSICHECK2.MCS. The synthetic DNA, which was constructed by annealing two oligonucleotides (Table 1, B) contained additional restriction sites (*Sal*I-*Sac*I-*Spe*I-*Age*I-*Xho*I-*Not*I) for direct subcloning of target 3’UTRs under Renilla luciferase coding sequence. pSGG-MSI1 plasmid was gifted from Dr. Luiz O.F. Penalva (UT Health San Antonio, USA) and digested with *Nhe*I and *Xho*I restriction enzymes. The resulted wild type 3′-UTR fragment of *MSI*I was ligated between *Spe*I and *Xho*I sites of pSICHECK2.MCS plasmid. Verification of final plasmid, pSICHECK2.MCS.MSI1, was performed through double digestion with *Xho*I and *Nhe*I enzymes and single digestion with *Xho*I. Restriction digestion patterns confirmed the plasmid size (Supplementary figure 8). Mutated type of MSI1 3’UTR for each miRNA binding site was constructed with site directed mutagenesis protocol [42] (Table 1, C) and these mutated fragments were cloned into pSICHECK2.MCS plasmid. Schematic representation of recombinant plasmids for luciferase assay were shown in Supplementary figure 9. The constructed vectors were named *MSI1*3’UTR (miR-125b).pSICHECK2, *MSI13*’UTR (miR-637).pSICHECK2 and *MSI1*3’UTR (miR-802).pSICHECK2. To confirm the sequence of the cloned fragments, final recombinant constructs were sent for sequencing by Metabion Company (Germany). Primers of Table 1, A and C were designed by oligo7.

**Table 1:** List of primers used in this study

**A) Primers used for human miRNAs precursors amplification**				
**Genes**	**Forward Primer (5′-3′)**	**Reveres Primer (5′-3′)**		
**miR-125b**	**GTCGACTGTGAAGGAAAGGAGC**	**TCTAGAACTTTAACAGAAATCCAGG**		
**miR-637**	**GTCGACACCCCAGGGATGATG**	**TCTAGA AGGCGGAGCACAGAAG**		
**miR-802**	**GTCGACCAGTTTGTTCCAGTGCC**	**TCTAGATAACACACACAGCCCCAG**		
**Scramble**	**GTCGACCCGCTTGTTCGTTGGTAACTACATTCAAGAGATGTAGTTACC**	**GTCTAGAAAAAAGCTTGTTCGTTGGTAACTACATCTCTTGAATGTAGTTACC**		
**B) Oligonucleotide sequences used for construction of pSICHECK2.MCS**				
**Name**	**(5′to3′)**			
**OligoF**	**TCGACCGGAGCTCATACTAGTACACCGGTAACTCGAGTAGC**			
**OligoR**	**GGCCGCTACTCGAGTTACCGGTGTACTAGTATGAGCTCCGG**			
**C) Primers used for site-directed mutagenesis PCR**				
**Mutated site**	**Forward Primer (5′-3′)**	**Reveres Primer (5′-3′)**		
**miR-125b**	**F2:GCCCGCTCTCTCTTGGTTGGACCTGC** **F3:AAAGCAAAGGGCGTACCCCCACATTCTCTfC**	**R2:GTCCAACCAAGGGCCTGAGAGAGCG** **R3:GAATGTGGGGGTACGCCCTTTGCTTTCC**		
**miR-637**	**F2:CTTGGCAGCCCCTCACCCCCCTTC**	**R2:GCCTCATAAAGAGGGACACACAGAAG**		
**miR-802**	**F2:CCTCAGACACCGTAAGCTTGCAGGCCTCAG** **F3:GCAATAATCTTTGAGATGCGCGGCTGTTC**	**R2:TTCACTCCAGCTATGCAC** **R3:GAACAGCCGCGCATCTCAAAGATTATTGCTTTGTG**		
**Common primer**	**F1:GTCGACAGCGCCCCAGCCTGCA**	**R1:CTCGAGAACGTTTTCAAATAATTTATTAGG**		
**D) Primers used for gene expression analysis by Q-PCR**				
**Genes**	**Forward Primer (5′-3′)**	**Reveres Primer (5′-3′)**	**TM (°C)**	**Accession No**
***MSI1***	**TTGGCAGACTACGCAGGAAG**	**TGGTCCATGAAAGTGACGAAGC**	**59**	***NM_002442***
***Gapdh***	**CCACTCCTCCACCTTTGACG**	**CCACCACCCTGTTGCTGTAG**	**60**	***NM_001357943***
***E-cadherin***	**CTGCTGCTCTTGCTGTTTCTTC**	**CTTCTCCGCCTCCTTCTTCATC**	**60**	***NM_001317185***
***EpCAM***	**CCATGTGCTGGTGTGTGAAC**	**CCTTCTGAAGTGGTCCGC**	**60**	***NM_002354***
***CD24***	**ATGTGGCAAGGAAAAACAGG**	**TTGGCATCCATCATCTAGTC**	**60**	***NM_001359084***
***Vimentin***	**AAACTTAGGGGCGCTCTTGT**	**TGAGGGCTCCTAGCGGTTTA**	**59**	***NM_003380***
***Snail***	**CCAGAGTTTACCTTCCAGCA**	**GATGAGCATTGGCAGCGA**	**60**	***NM_005985***

### Cell culture

HEK293T and breast cancer cells (T-47D, MCF-7, SK-BR-3 and MBA-MD-231) were purchased from the Pasteur Institute (Tehran, Iran). Cell culture was performed in 10% FBS supplemented DMEM high Glucose (Gibco, USA) including 100 units/mL penicillin and 100 μg/mL streptomycin (Gibco, 15,070,063) as well as 2 mM L-glutamine (Gibco, 25,030–024), 1% non-essential amino acids (Gibco, 11,140–035). Normal breast cell line, MCF-10A, was cultured in DMEM/F12 (Gibco, 21,331–020) supplemented with 5% horse serum (ATOCEL, Aths-8100), insulin 10 μg/mL (Sigma, 16,334), cholera toxin (Sigma, c8052) 100 ng/mL, hydrocortisone (Sigma, H0888) 0.5 mg/mL, EGF (Sigma, 4127) 20 ng/mL, 100 units/mL penicillin and 100 μg/mL streptomycin (Gibco, 15,070,063), 2 mM L-glutamine (Gibco, 25,030–024). Cell culture was performed under 5% CO_2_ at 37 °C.

### Transcript analysis

The extraction of total RNA was achieved using TRI reagent (Thermo Scientific, 15,596–018) and cDNAs were synthesized using random hexamer primers and implementing MMLV reverse transcriptase according to the manufacturer’s protocol (TaKaRa, RR037A, Japan). To get rid of DNA contamination, DNase treatment was performed during cDNA synthesis. At the next step, 50 ng of cDNA was used for real-time PCR using SYBR green (TaKaRa, RR820Q, Japan) and specific primers on step one plus Real-Time PCR System (Applied Biosystems, USA). Primers were designed by the Beacon designer (Version 7.2, USA) and purchased from Metabion Company (Table 1, D). *Glyceraldehyde 3-phosphate dehydrogenase* (*Gapdh*) was used as reference gene for normalizing target gene expression and each experiment was repeated thrice. For microRNAs expression, cDNAs were synthesized using Universal cDNA synthesis kit II (Exicon, 203,301, Denmark). LNA™ primer mix for mature miRNAs and LNA™ Primer mix U6 (Exicon, Denmark) was used for Real time PCR. Finally, analysis of relative gene expression was performed using ΔΔCt method.

### Immunoblot analysis

Proteins were extracted by means of TRI reagent (Thermo Scientific, 15,596–018) according to manufacturer instructions. Similar amount of protein for each sample, approximately 30 μg, was separated using SDS-PAGE and transferred to polyvinylidenedifluoride (PVDF; Bio Rad, 162–0176, USA) membranes. Blocking of the membranes was done in skim milk 10% (Millipore, 115,363) and then membranes were incubated with specific primary antibodies at 25 °C for 1.5 h. Primary antibodies were Anti-MSI1 antibody (Abcam, 52,865, USA; 1:4000), and mouse anti-Gapdh, clone 6C5 (Millipore, MAB374, USA; 1:5000). After this step, membranes were incubated at the same condition for the respective secondary antibodies, which were horseradish peroxidase (HRP)-conjugated a) goat anti-mouse IgG (Dako, P0447, USA; 1:5000) or b) goat anti-rabbit IgG (Santa Cruz, SC2301, USA; 1:16000). The HRP-conjugated IgG bound to each protein band was visualized by an Amersham ECL Advance Western Blotting Detection Kit (GE Healthcare, Germany).

### Luciferase reporter assay

For Luciferase assay, HEK293T were seeded in 24-well plate to reach enough confluence for 24 h. Then, cells were co-transfected with pSICHECK2.MCS.MSI1 and each of precursor expressing plasmid. Besides, plasmid contained mutated type of MSI1 3’UTR for each miRNA binding site along with precursor expressing plasmid were co-transfected into HEK293T cells. Co-transfections were accomplished using Lipofectamine 2000 (Invitrogen, USA) according to its protocol. Approximately 2 days post-transfection, the cells were harvested and analyzed with dual-Luciferase reporter assay system (Promega, USA).

### Immunofluorescence analysis

Approximately 48 h post-transfection, miR-125b.pBUD transfected MCF-7 cells were treated with 4% paraformaldehyde for 30 min at 25 °C. Permeabilization of the cells were achieved through incubating with 0.4% Triton X-100 for 20 min. After cell washing, primary antibody was diluted by blocking solution (BSA, 5 mg/mL) and used for cell staining. The cells were then incubated at 4 °C overnight. In the next step, the cells were stained with secondary antibody for 1 h at 37 °C and finally, they were stained with DAPI (D9542, Sigma) at final concentration of 1 μg/mL at room temperature for 5 min. Both first and secondary antibodies were Musashi-1 antibody (1:100, R&D: AF2628) and goat anti-rabbit IgG-FITC (1:100, Santa Cruz: Sc-2012). Images were captured under a fluorescent microscope (Olympus, Center Valley, PA, USA) using Olympus DP70 camera. CD24 expression was assayed using flow cytometry. Co-transfected MCF-7 cells with miR-125b.pBUD and pBUD.USc were detached using triple (Gibco, 12,604–012) after 48 h. Treated cells were stained with PE labeled mouse Anti-Human CD24 (BD Pharmingen™, 555,428, USA) for 30 min. After washing, the cells were evaluated by FACS Caliber flow cytometer (Becton Dickinson, USA and obtained results were analyzed with the ModFit LT™ v 4.0 program.

### Statistical analysis

SPSS Statistical software (v. 20, IBM Corp., USA) was used to perform the data analysis and all data were reported as mean ± standard error of mean (SEM). Independent student *t*-test and one-way analysis of variance (ANOVA) were utilized to determine the statistical differences between groups, where *p* < 0.01 and *p* < 0.05 have been applied for indication of statistically significant differences.

## Supplementary Information

**Additional file 1: Figure S1.** The results of GEO dataset GSE70480 for miRNAs selection. Related accession: GSM1782670 is for MCF-10A as normal breast cell line, GSM1782672 is for MCF-7 and GSM1782680 is for T-47D.

**Additional file 2: Table S1.** microRNA target sites in *MSI1* 3`UTR.

**Additional file 3: Figure S2.** MCF-7 cell line were transfected with miR-125b.pBUD and USc.pBUD expression plasmids. EGFP reporter gene indicated the accuracy of transfection.

**Additional file 4: Figure S3.** Reduction of MSI1 intensity in MCF-7 cell line which transfected with miR-125b.pBUD. The separated area demonstrated EGFP positive cells with decreased intensity of MSI1 as a result of overexpression of miR-125b.

**Additional file 5: Figure S4.** MSI1 intensity in MCF-7 cell line which transfected with (USc.pBUD) plasmid. Selected area shows EGFP positive and negative cells which have the same intensity for MSI1.

**Additional file 6: Figure S5.** Flow cytometry data confirmed reduction of MSI1 intensity in MCF-7 cell line after transfection with miR-125b.pBUD.

**Additional file 7: Figure S6.** Agarose electrophoresis of double digestion products of recombinant plasmids [pBUD/precursor miRNAs expression plasmids] which were digested with *Sal*I and *Xba*I restriction enzymes. Double digestion with *Sal*I and *Xba*I resulted in two distinct bands with approximate sizes of 5191 bp for plasmid pBUD and 378 bp for precursor of miR-637, 453 bp for precursor of miR-802 and 427 bp for precursor of miR-125b. M is 1kbp DNA ladder (Thermo Scientific, USA).

**Additional file 8: Figure S7.** Schematic representation of recombinant plasmids for ectopic expression of selected miRNAs which is involved miRNAs precursors and recombinant vector included scramble sequence as negative control.

**Additional file 9: Figure S8.** Agarose electrophoresis of double digestion products of recombinant plasmids [pSICHECK2.MCS.MSI1 plasmids] which were digested with *Xho*I and *Nhe*I restriction enzymes and single digestion with *Xho*I restriction enzyme. *Xho*I-linearized plasmid with a single band about 8053 bp and double digested plasmid fragments 5292 bp and 2761 bp. M is 1kbp DNA ladder (Thermo Scientific, USA).

**Additional file 10: Figure S9.** Schematic representation of pSICHECK2 plasmid which included MSI1 wild type and mutated MSI1 for direct binding of particular miRNAs.

## Data Availability

Supporting and raw data are available upon a reasonable request to the corresponding author.

## Abbreviations

CK2-α: Casein kinase 2-alpha
EGF: Epidermal growth factor
ENPEP: Amino peptidase A
RT-qPCR: Real-time quantitative polymerase chain reaction
CDS: Coding sequence
CCNJ: Cyclin J
JDMEM: Dulbecco’s modified Eagle medium
EMT: Epithelial-mesenchymal transition
FCS: Fetal calf serum
MEGF9: Multiple EGF-like-domains
M-MLV Reverse Transcriptase: Moloney Murine Leukemia Virus Reverse Transcriptase
GAPDH: Glyceraldehyde 3-phosphate dehydrogenase
HER2: Human epidermal growth factor receptor 2
HRP: Horseradish peroxidase
Msi1: Musashi1
PBS: Phosphate-buffered saline
PVDF: Polyvinylidenedifluoride
SEM: Standard error of mean
STAT3: Signal transducer and activator of transcription 3
TNBC: Triple negative breast cancer
TRI: Trizol
3`-UTR: 3`-untranslated region
