# Supplementary figures and images for: Increased expression of MUSASHI1 in epithelial breast cancer cells is due to down regulation of miR-125b
Source: BMC Mol Cell Biol. 2021 Feb 4;22:10. doi: 10.1186/s12860-021-00348-8 (PMC7863248; doi:10.1186/s12860-021-00348-8)

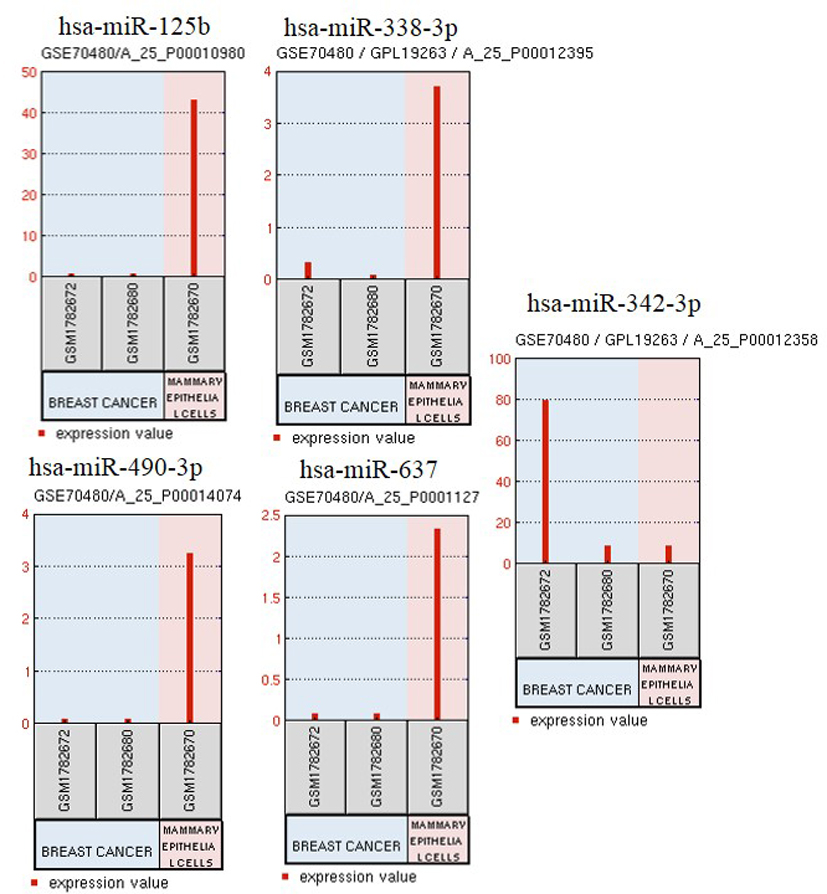

Supplement: Supplementary file 1 — Additional file 1: Figure S1. The results of GEO dataset GSE70480 for miRNAs selection. Related accession: GSM1782670 is for MCF-10A as normal breast cell line, GSM1782672 is for MCF-7 and GSM1782680 is for T-47D. [file 12860_2021_348_MOESM1_ESM.jpg]

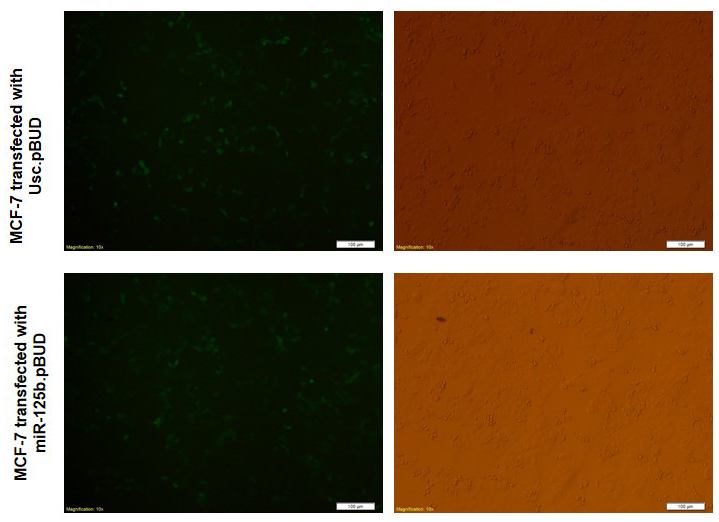

Supplement: Supplementary file 3 — Additional file 3: Figure S2. MCF-7 cell line were transfected with miR-125b.pBUD and USc.pBUD expression plasmids. EGFP reporter gene indicated the accuracy of transfection. [file 12860_2021_348_MOESM3_ESM.jpg]

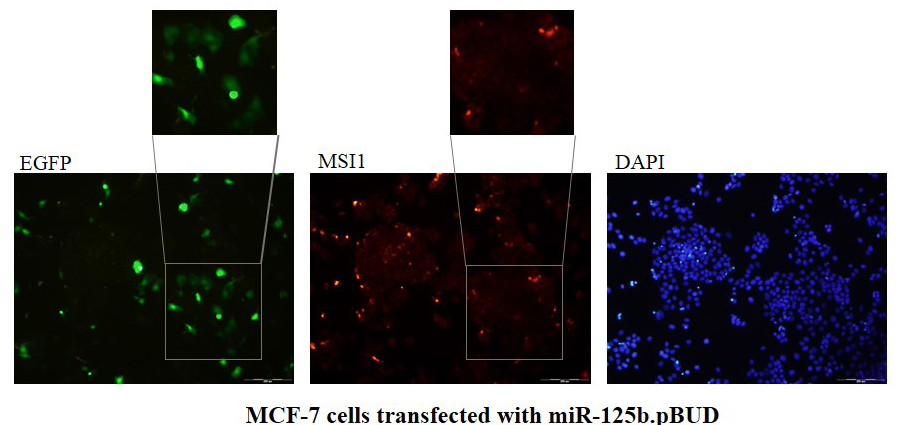

Supplement: Supplementary file 4 — Additional file 4: Figure S3. Reduction of MSI1 intensity in MCF-7 cell line which transfected with miR-125b.pBUD. The separated area demonstrated EGFP positive cells with decreased intensity of MSI1 as a result of overexpression of miR-125b. [file 12860_2021_348_MOESM4_ESM.jpg]

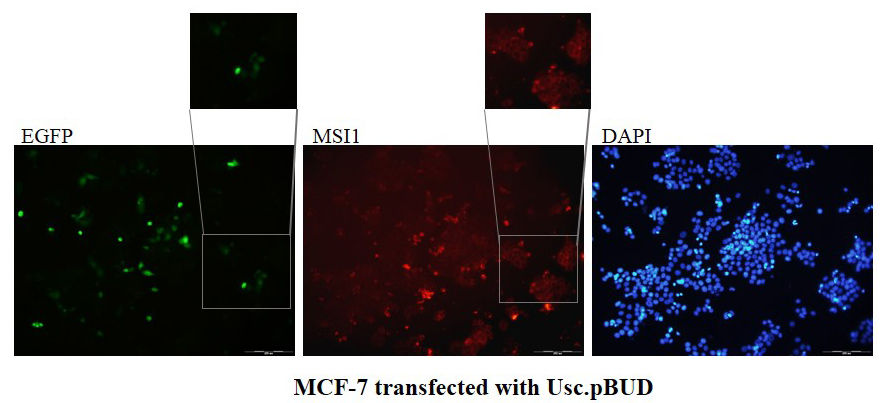

Supplement: Supplementary file 5 — Additional file 5: Figure S4. MSI1 intensity in MCF-7 cell line which transfected with (USc.pBUD) plasmid. Selected area shows EGFP positive and negative cells which have the same intensity for MSI1. [file 12860_2021_348_MOESM5_ESM.jpg]

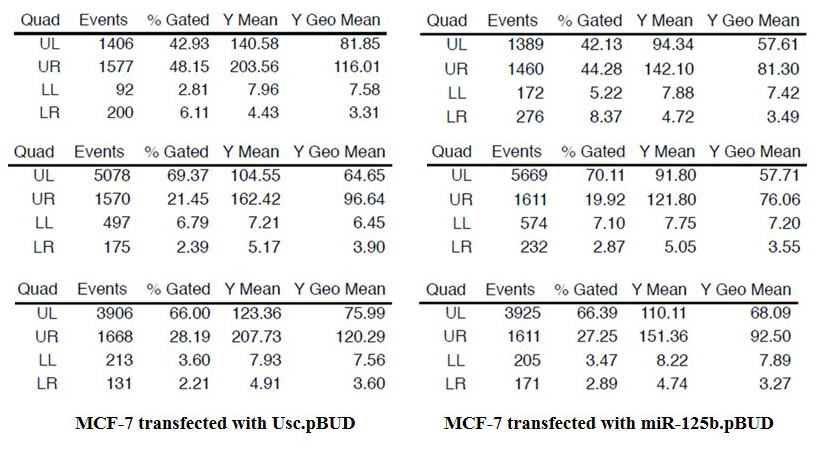

Supplement: Supplementary file 6 — Additional file 6: Figure S5. Flow cytometry data confirmed reduction of MSI1 intensity in MCF-7 cell line after transfection with miR-125b.pBUD. [file 12860_2021_348_MOESM6_ESM.jpg]

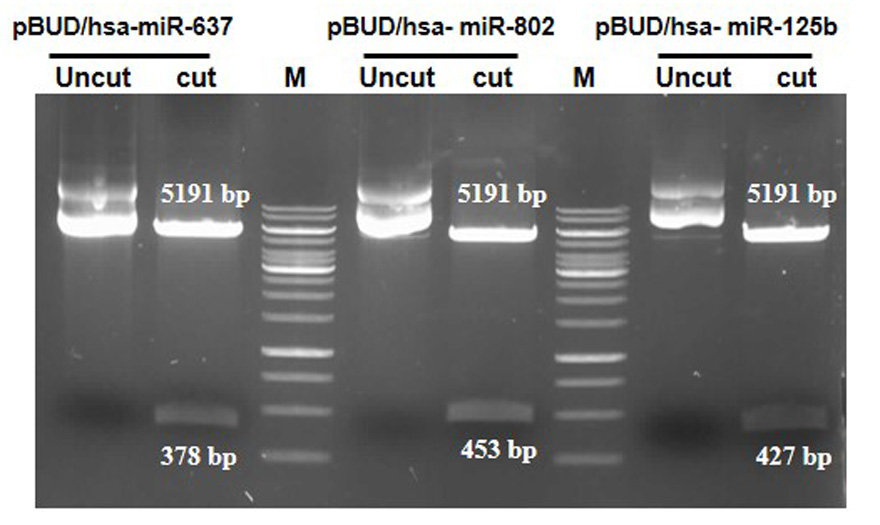

Supplement: Supplementary file 7 — Additional file 7: Figure S6. Agarose electrophoresis of double digestion products of recombinant plasmids [pBUD/precursor miRNAs expression plasmids] which were digested with SalI and XbaI restriction enzymes. Double digestion with SalI and XbaI resulted in two distinct bands with approximate sizes of 5191 bp for plasmid pBUD and 378 bp for precursor of miR-637, 453 bp for precursor of miR-802 and 427 bp for precursor of miR-125b. M is 1kbp DNA ladder (Thermo Scientific, USA). [file 12860_2021_348_MOESM7_ESM.jpg]

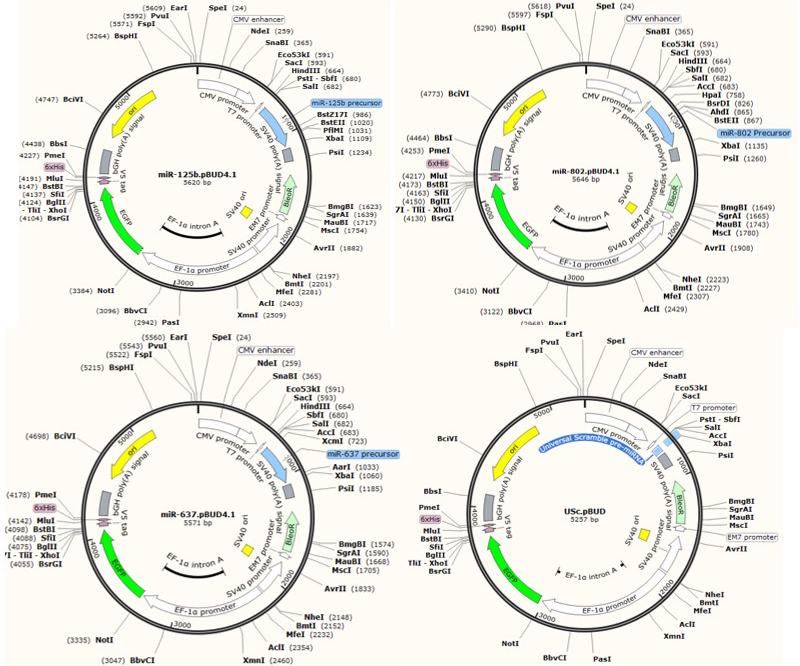

Supplement: Supplementary file 8 — Additional file 8: Figure S7. Schematic representation of recombinant plasmids for ectopic expression of selected miRNAs which is involved miRNAs precursors and recombinant vector included scramble sequence as negative control. [file 12860_2021_348_MOESM8_ESM.jpg]

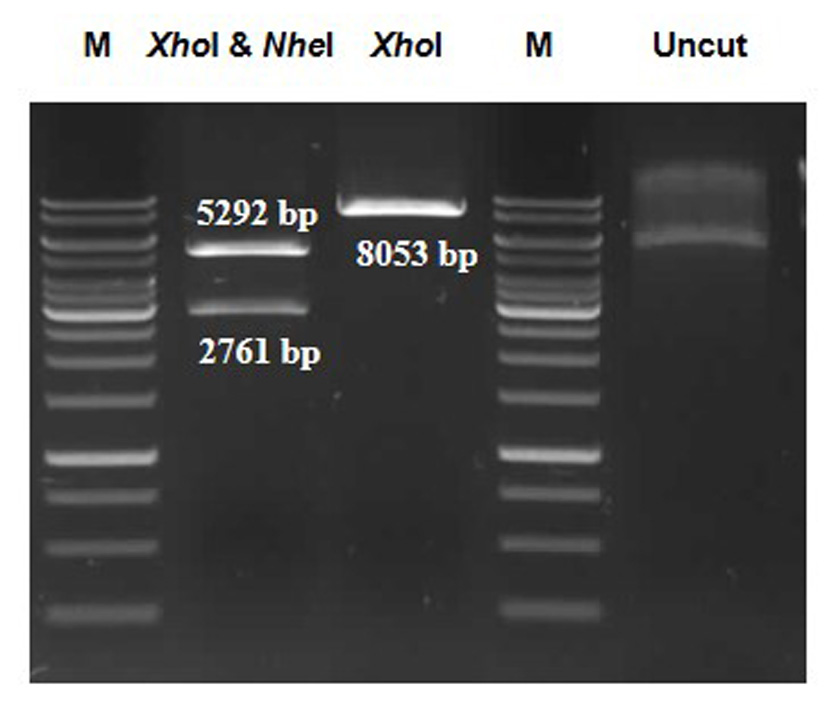

Supplement: Supplementary file 9 — Additional file 9: Figure S8. Agarose electrophoresis of double digestion products of recombinant plasmids [pSICHECK2.MCS.MSI1 plasmids] which were digested with XhoI and NheI restriction enzymes and single digestion with XhoI restriction enzyme. XhoI-linearized plasmid with a single band about 8053 bp and double digested plasmid fragments 5292 bp and 2761 bp. M is 1kbp DNA ladder (Thermo Scientific, USA). [file 12860_2021_348_MOESM9_ESM.jpg]

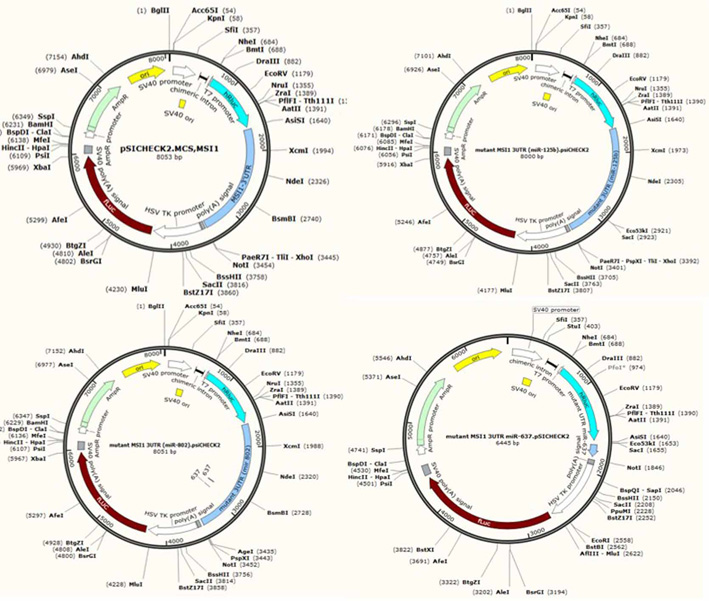

Supplement: Supplementary file 10 — Additional file 10: Figure S9. Schematic representation of pSICHECK2 plasmid which included MSI1 wild type and mutated MSI1 for direct binding of particular miRNAs. [file 12860_2021_348_MOESM10_ESM.jpg]
