# Supplementary material for: Increased expression of MUSASHI1 in epithelial breast cancer cells is due to down regulation of miR-125b
Source: BMC Mol Cell Biol. 2021 Feb 4;22:10. doi: 10.1186/s12860-021-00348-8 (PMC7863248; doi:10.1186/s12860-021-00348-8)
Supplement: Supplementary file 2 — Additional file 2: Table S1. microRNA target sites in MSI1 3`UTR. [file 12860_2021_348_MOESM2_ESM.docx]

**Supplementary table 1: microRNA target sites in *MSI1* 3`UTR**

| **The miRNAs Name** | **Predicted consequential pairing of 3`UTR region of *MSI* (top) and miRNAs (bottom)** | **Position of *MSI1* 3`UTR** |
| --- | --- | --- |
| **miR-125b** | 5'  ...GAAAGCAAAGGGCGU**CUCAGG**GA...                        **\| \| \| \| \| \|** 3'       AGUGUUCAAUCCCA**GAGUCC**CU | **Position 1090-1097** |
|  | 5' ...CCGCUCUCUCAGGCC**CUCAGGG**U...                        **\| \| \| \| \| \| \|** 3'     AGUGUUCAAUCCCA**GAGUCCC**U | **Position 834-840** |
| **miR-637** | 5'  ...CUUGGCAGCCCCUCA-----**CCCCAGA**C...              \| \| \| \| \|     **\| \| \| \| \| \|**   3'  UGCGUCUCGGGCUUUCGG**GGGUC**A | **Position 1169-1175** |
|  | 5' ...UCUGUGUGUCCCUCU------C**CCCCAG**U...                  \| \| \|    **\|**  **\|**  **\| \| \| \| \|**  3'   UGCGUCUCGGGCUUUCG**GGGGUC**A | **Position 1292-1298** |
| **miR-802** | 5'   ...GUCCCCUCAGACACC**GUUACUG**U... **\| \| \| \| \| \|  \|** 3'  UGUUCCUACUUAGAAA**CAAUGAC**U | **Position 777-783** |
|  | 5'  ....AAAGCAAUAAUCUUU**GUUACUG**A...  **\| \| \| \| \| \| \|**  3'   UGUUCCUACUUAGAAA**CAAUGAC**U | **Position 1428-1435** |
